# Ovipositional periodicity of caged *Anopheles gambiae *individuals

**DOI:** 10.1186/1740-3391-6-2

**Published:** 2008-01-25

**Authors:** Megan L Fritz, Juan Huang, Edward D Walker, M Nabie Bayoh, John Vulule, James R Miller

**Affiliations:** 1 Department of Entomology, Michigan State University, 203 Center for Integrated Plant Systems, East Lansing, MI, 48824, USA; 2 Department of Microbiology and Molecular Genetics, Michigan State University, 6169 Biomedical Physical Sciences Building, East Lansing, MI, 48824, USA; 3 Centre for Global Health Research, Kenya Medical Research Institute (KEMRI), P.O. Box 1578, Kisumu, Kenya

## Abstract

**Background:**

*Anopheles gambiae s.s*. Giles is a major malaria vector in Sub-Saharan Africa. Studies of the basic biology of this mosquito, including oviposition, provide a background for assessing which attributes might be exploited for suppressing *A. gambiae *populations. Here, we report on when during the diel cycle *A. gambiae *individuals deposit eggs as compared to the ovipositional patterns of groups.

**Methods:**

Battery-powered wall clocks were modified so as to present a unique section of dark and wet ovipositional substrate at hourly intervals over two consecutive 12 h periods. Ovipositional periodicity of mosquito groups (Kisumu laboratory strain or feral females) and individuals was determined by counting the number of eggs present on each section of the ovipositional substrate. Capacity for mid-afternoon oviposition by groups of Kisumu laboratory strain *A. gambiae *was determined by presenting hypergravid females with an ovipositional substrate exclusively between 1200 and 1600 h.

**Results:**

On equatorial time, caged laboratory strain *A. gambiae *groups deposited 65% of their total eggs between 1800 and 0 h, and the remaining 35% were spread between 0 and 1000 h. Caged house-collected *A. gambiae *groups deposited 74% of their total eggs between 1800 and 200 h, ceased oviposition for 3 h, and then spread the remaining 26% of their eggs near or after dawn. Ninety-six percent of individual *A. gambiae *females spread their eggs over a continuous 2–4 h period without interruption. In tests of capacity for mid-afternoon oviposition, females given evening access to an ovipositional resource deposited 2% of their total eggs between 1200 and 1700 h. *A. gambiae *females given only access to an ovipositional resource between 1200 and 1700 h deposited 3 times more eggs during that time period than did females previously given evening access.

**Conclusion:**

Confined individual *A. gambiae *oviposit in a single ca. 2–4 h continuous bout per 24 h. Oviposition is most probable in early scotophase, mid scotophase, or early photophase. However, some oviposition can occur at any hour during 24 h, especially if females were previously deprived of ovipositional substrate.

## Background

Diel ovipositional patterns have been studied in various Diptera, including *Drosophila melanogaster *[1-4], *D. pseudoobscura *[5], *Delia antiqua *[6], *Chrysomya bezziana *[7], *Aedes aegypti *[8], *Anopheles albimanus *[9], *A. freeborni *[9], *A. albitarsis *[10] and *A. gambiae *[11-14]. All of these species are reported to deposit the preponderance of their forthcoming eggs within a 2–4 h period [1-14], but the time of maximal egg deposition varies interspecifically. However, in no case was oviposition reported to occur strictly within that 2–4 hour window. For *A. albimanus *[9] and *D. melanogaster *[4], ovipositional rhythm was reported to be bimodal with unequal modes; light intensity during photophase was reported to influence the modality of *D. melanogaster *oviposition [3]. Fluegel [4] found that light levels furnished by a 40 W white fluorescent bulb resulted in bimodal egg deposition by *D. melanogaster *individuals. Chadee et al. [9] reported that individual *A. albimanus *laid their entire complement of eggs at once rather than splitting them between two different periods.

Outcomes of research on the ovipositional periodicity of groups of *A. gambiae *held in a common cage [11-14] have been divergent. Causey et al. [11] suggested that *A. gambiae *was capable of oviposition at any time during the night. However, they observed that five of a total of nine batches of eggs were laid between 2000 and 2300 h. Under equatorial conditions, McCrae [13] reported wide variation in nocturnal peaks for *A. gambiae *oviposition. He postulated that the time of peak oviposition during a night was related to the time at which the blood meal was taken. However, Sumba et al. [14] were unable to confirm this effect. Haddow and Ssenkubuge [12] and Sumba et al. [14], reported that oviposition of *A. gambiae *commenced at scotophase (1800 h) and peaked between 1800–2100 h. A second but smaller ovipositional peak was documented by both research teams, but at inconsistent times. In all cases, some oviposition occurred throughout scotophase. In one case [14], a feral population deposited about 3% of the total eggs after the onset of photophase. In other cases, it was unclear whether any attention was paid to the possibility of oviposition throughout photophase. Left unknown in all of these studies is whether individual females spread their oviposition across many hours, or whether some individuals deposit all of their eggs early in the night while others deposit all of their eggs near morning.

## Methods

### Mosquitoes

Two sources of mosquitoes were used: 1) The Kisumu laboratory strain of *A. gambiae s.s*. originated from the Kenya Medical Research Institute (KEMRI) of Kisumu, Kenya. It was reared at Michigan State University according to Huang et al. [15]. Cages of mosquitoes were held in an environmental chamber maintained at 28 ± 1°C and 80 ± 10% RH under a LD 12:12 h photoperiod. Indirect light of about 0.17 lx was provided during scotophase by a shaded 4 watt tungsten bulb; it was intended to mimic moonlight. Mosquitoes were offered blood via a membrane feeder 2–3 days before ovipositional tests. 2) Blood-fed feral females were aspirated from the walls of houses near the KEMRI compound. These females were held in laboratory cages under high humidity for 24 h before ovipositional tests. As previously reported, ca. 90% of the females were *A. gambiae *s.s. and the remainder were *A. arabiensis *as determined by PCR [15].

### Automated Ovipositional Clock

The method of egg collection may influence the oviposition rhythm of some Diptera [16]. Use of a mechanized egg collector may be less disruptive to egg deposition than manually changing an ovipositional resource at hourly or two-hour intervals. Mechanized egg collectors have previously been utilized in the study of ovipositional rhythms for both *D. melanogaster *[5,16] and *Agrotis segetum *[17]. In the current study, we developed a new automated mechanical apparatus to sample oviposition over time. This apparatus was used to compare ovipositional periodicity by *A. gambiae *individuals vs. groups.

**Figure 1:**
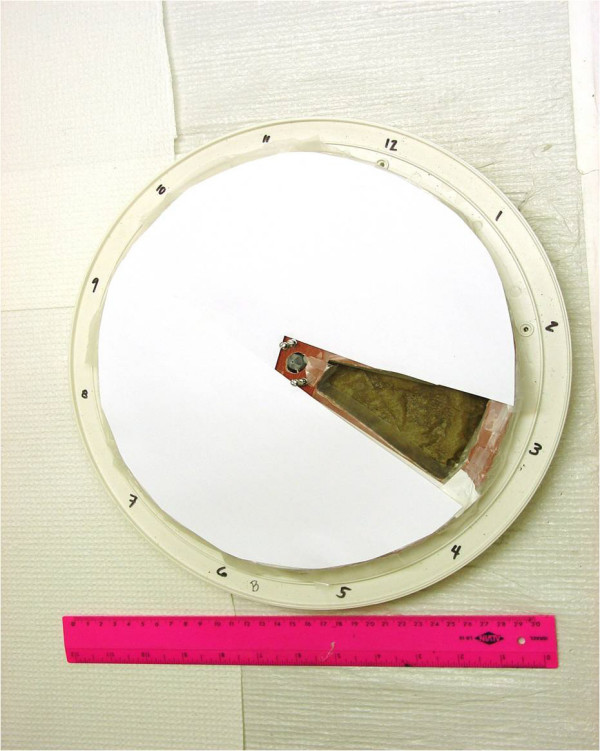
Clock apparatus used in automated measurement of *Anopheles gambiae *ovipositional periodicity.

Battery-powered wall clocks (DSA Incorporated, Farmington Hills, MI, U.S.A.) measuring 31 cm in diameter were modified to progressively present a unique section of dark and wet substrate onto which mosquitoes could oviposit over 12 h (Figure 1). Each clock was positioned horizontally and its original face and hands were removed. The clock body was filled with moist sand of particle size 250 – 425 μm. The sand was topped with Envision^® ^high-capacity brown paper towelling (Georgia Pacific, Camas, WA, U.S.A). This paper towelling appears light when dry, but dark when wet. Thus, it provided the two key stimuli (dark and wet) necessary and sufficient to strongly stimulate *A. gambiae *to oviposit [15]. A new and removable clock face was fashioned from a circular piece of thin plastic, from which had been cut a section equivalent to one hour on the clock (Figure 1). The opening and perimeter of the face-plate were lined with Parafilm ^® ^(Pechiney Plastic Packaging, Menasha, WI, U.S.A.) flaps to prevent mosquitoes from depositing eggs on any unexposed section of the substrate. The clock face was mounted on the hour-hand driver, so that the opening in the face plate made one revolution every 12 h. The clock face was covered with white paper so as to maximize contrast between background versus the actual ovipositional site [18].

Prior to insertion into the clock apparatus, the paper towel substrate was divided by pencil marks into 12 equal wedge-shaped sections. After being exposed to gravid female mosquitoes for 12 h, a clock was removed from the cage of mosquitoes and another was immediately inserted so as to extend the study over a full 24 h. The face of an exposed clock was carefully removed and the paper towelling bearing eggs was carefully peeled off the sand for egg counting under a dissecting microscope. Clock sections open at the beginning and end of a given measurement were exposed to females for a total of 1 h. However, it took 1 h for each intervening section to fully open and another 1 h for each to fully close. Thus, some of the eggs on each intervening section could have been laid over a span of 2 h.

### Experimental Series 1 – Automated measurement of caged mosquito groups

Clocks were presented in white BugDorm-2 insect rearing cages (Mega View Science Education Services Co., Taiwan) measuring 60 × 60 × 60 cm and containing approximately 500 laboratory-reared females of the Kisumu strain varying in reproductive stages. The light cycle in the environmental chamber was set at 12:12 LD, to approximate the natural light cycle found in Kisumu, Kenya. A small tungsten bulb continued to burn in the laboratory at night so as to provide the equivalent light from the night sky. Light levels during scotophase were slightly less than full moonlight (10^-3 ^W m^-2^; [19]). Ovipositional clocks were also presented to groups of house-collected gravid females as described above. In this experiment, the BugDorm-2 cages housing approximately 100 females were placed just inside a screened porch of a house in Kisumu, Kenya. Egg recording sessions for both house-collected and laboratory reared groups were replicated 8 and 6 times respectively, using a different set of females for each test. Each recording session began at 1700 h, one hour prior to the onset of scotophase, and continued for 24 h. At 500 h, a clock apparatus containing fresh paper towelling was exchanged for the loaded clock. The numbers of eggs laid within each hourly period were counted under a dissecting microscope and incorporated into frequency histograms.

### Experiment 2 – Automated measurement of caged individuals

The bottom of the enclosure for these tests was the clock apparatus over which sat a 12 cm high cylindrical wire frame. Nylon netting (18 intersections/cm) was placed over the frame and secured by a drawstring. Six 2 cm diameter wet cotton balls (Kendall, Mansfield, MA) were placed on the roof of the cage as a source of moisture. Blood meals were offered to females between 1200 and 1700 h three to four days prior to use. Three or four days after a blood meal, an individual female was gently transferred to the clock cage by aspirator before scotophase. After the female had been exposed to the ovipositional resource for 4–5 h, the female was removed from the cage and fresh paper towelling was substituted for the previously exposed paper towelling within the clock. Then, the female was carefully reinserted. The exchange of the ovipositional resource was repeated 11 h later. The numbers of eggs laid within each hourly period were counted and incorporated into a histogram. Correlation analysis (SAS software version 9.1) was used to test for a correlation between the length of the preoviposition interval (defined as the time interval between a female's first exposure to the clock and the time oviposition was initiated) and the length of the oviposition interval (time interval during which oviposition occurred). It was also used to test for a correlation between the length of the preoviposition interval and the total number of eggs deposited per female. The ovipositional periodicity was measured for a total of 56 individual females, all of the Kisumu laboratory strain.

The terms gravid and hypergravid as used by Sumba et al. [14] refer the condition of the female mosquito when they are presented with an ovipositional resource three and four days, respectively, after obtaining a blood meal. Differences in oviposition by gravid versus hypergravid females were examined by comparing the mean numbers of eggs oviposited per female per hour of each respective group using a paired t-test (SAS software version 9.1). After each trial, females were dissected under a dissecting microscope to check for residual eggs.

### Experiment 3 – Assessment of capacity for mid-afternoon oviposition

Engorged females were randomly selected from newly blood-fed cages of mosquitoes and placed in groups of 20 into 8 cages made from 15 cm high and 19 cm in diameter white cardboard cartons. The top of the cage was covered with white netting (8 intersections/cm) and a sleeve of the same netting was fitted to a 10.5 cm hole cut in the side for mosquito and ovipositional resource insertion. Females were provided with a constant source of 10% honey solution and six wet cotton balls (Kendall, Mansfield, MA) were placed on the top of the cage to provide extra moisture. Two days after blood-feeding, an ovipositional resource was provided to half of the cages approximately 2 h before the lights were turned off to record egg deposition during scotophase. The ovipositional resource was a 100 × 35 mm clear plastic Petri dish containing 20 mL of distilled water, placed over a circular piece of black paper. At 1200 h the following day, the loaded ovipositional resources were replaced with new Petri dishes containing fresh filtered water. Four ovipositional resources were also introduced into the 4 cages from which an ovipositional resource had been withheld. These resources, identical to those previously mentioned, were used to record oviposition by gravid females during photophase. After the initial introduction, ovipositional resources were changed hourly from 1200–1600 h in the latter half of the cages and all exposed ovipositional resources were examined for the presence of eggs. Using a small brush, eggs present were brushed into lines on a piece of white paper and counted.

## Results

### Experimental Series 1 – Ovipositional periodicity of caged groups

**Figure 2:**
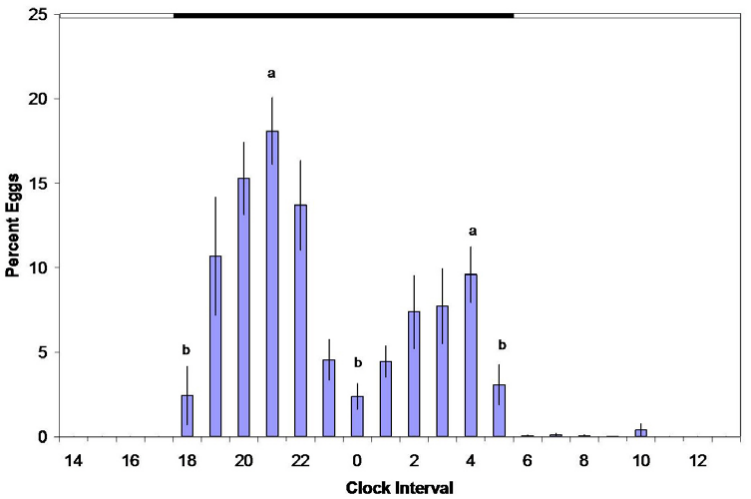
**Ovipositional periodicity of laboratory strain groups**. Mean percent of eggs oviposited per hour by a caged laboratory strain group (500 females per replicate; total eggs = 18,303).

*A. gambiae *of the Kisumu laboratory strain revealed two ovipositional pulses (Figure 2). The first occurred from 1800 to 0 h, peaked at 2100 to 2200 h, and accounted for 65% of the total eggs deposited. A second but smaller pulse occurred between 0 and 1000 h, and peaked at 400 h. It is notable that some oviposition by females in groups occurred throughout scotophase. Moreover, a few eggs were deposited in the early hours of photophase. The percent eggs deposited by the laboratory strain during the first peak (2100 h) was significantly greater than the percent eggs deposited at 0 h when oviposition greatly diminished (p < 0.0001; Tukey's HSD test). The proportion of eggs deposited during the second peak was not significantly different from the proportion deposited during the first peak at 2100 h (p = 0.3). The valley between these two peaks was marginally significant (p = 0.054).

**Figure 3:**
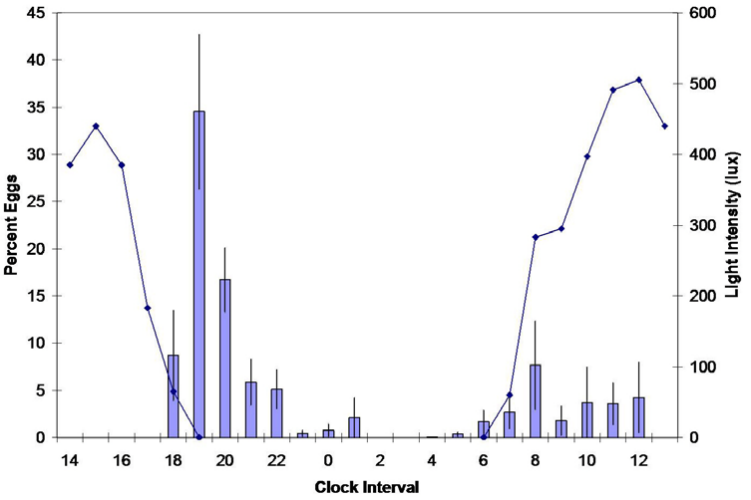
**Ovipositional periodicity of house-collected groups**. Mean percent of eggs oviposited per hour by caged house-collected groups (100 females per replicate; total eggs = 11,007). Measurements of outdoor light intensity represented in the graph as a dark blue line, were taken on 5/11/2004 in Kisumu, Kenya.

Two discrete pulses of oviposition were recorded for house-collected *A. gambiae *groups (Figure 3). The first began at dusk, peaked between 1900 and 2000 h, and ceased after 100 h. Seventy-four percent of the total eggs were laid between 1800 and 200 h. The second pulse commenced near dawn, peaked around 800 h, and ceased before 1300 h. Unlike the laboratory strain, wild-caught females deposited a substantial portion (more than 25%) of their eggs after sunrise.

### Experiment 2 – Ovipositional periodicity of caged individuals

Ovipositional periodicity of gravid and hypergravid females held individually was similar, although more gravid females contributed to the second ovipositional pulse than did hypergravids. A paired t-test of the total mean number of eggs oviposited per *A. gambiae *female per each hr (1700–1900 and 2100–2200 h) revealed no significant difference between gravid and hypergravid treatments (p = 0.61). However, correlation analysis revealed a significant positive correlation between the length of the preoviposition interval and the total number of eggs deposited (p = 0.03).

**Figure 4:**
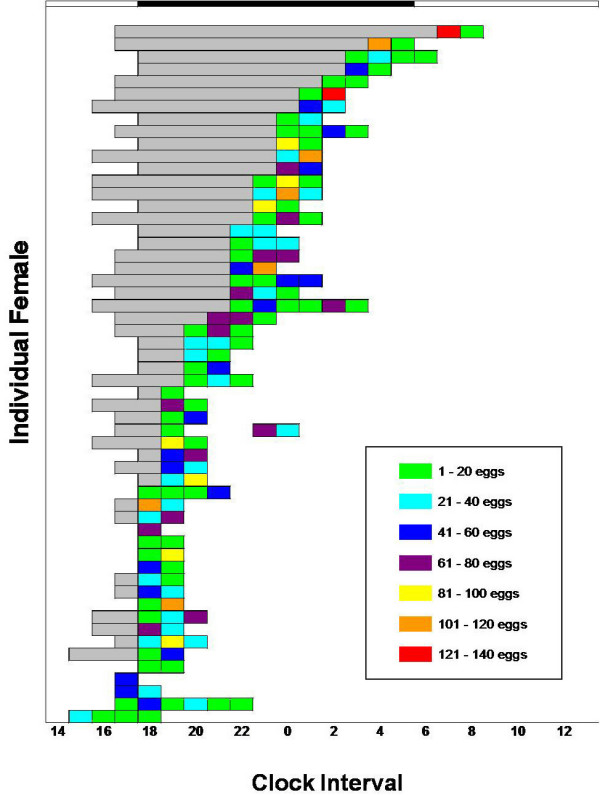
**Ovipositional patterns of individual *A. gambiae *over 24 h**. Each horizontal cluster of rectangles represents a single individual. Shading classifies the number of eggs deposited per individual per hr (n = 56) during the oviposition interval. Gray shading represents the preoviposition interval.

**Figure 5:**
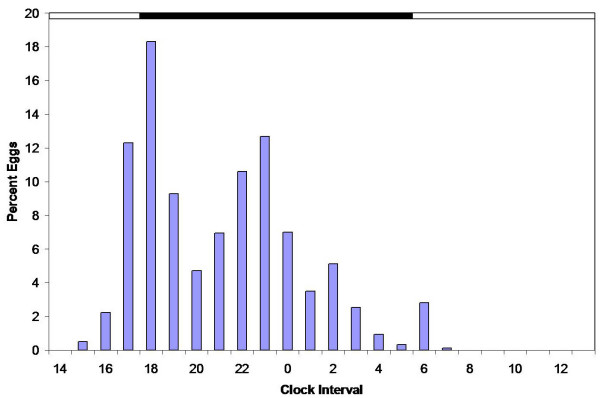
**Accumulated ovipositional patterns of individual *A. gambiae *over 24 h**. Percent of eggs oviposited per hour by caged individual *A. gambiae *(total eggs = 4,815).

The time at which individual females initiated oviposition was highly variable (Figure 4). The mean length of the preoviposition interval was 3.5 h with a variance of 9.5, and the mean length of the oviposition interval was 2.5 h with a variance of 1.0. A Levene's test, as modified by Brown and Forsythe, was used to compare the variances of the preoviposition and oviposition intervals; it yielded a p-value of < 0.0001. However, there was no correlation between the length of the preoviposition interval and the length of the oviposition interval (p = 0.51). Compiled individual oviposition was similar to patterns of groups (fig. 5); egg deposition occurred throughout scotophase and even during certain hours of photophase. Seven percent of individuals commenced egg deposition before lights off and one individual initiated oviposition after lights on. Interestingly, Figure 4 documents that most females oviposited without detectable interruption and those females spread their eggs continuously over a few consecutive clock intervals. Only one individual out of 56 exhibited two ovipositional pulses; she commenced at 1900 h, ovipositing 12 eggs, and then paused until 2300 h before depositing another 105 eggs.

### Experiment 3 – Assessment of capacity for mid-afternoon oviposition

Seven out of the 12 cages from which an ovipositional resource had been withheld until mid-afternoon produced eggs (Table 1). All of the 12 cages provided with an evening ovipositional resource produced eggs. Cages provided only with a mid-afternoon ovipositional resource produced 641 total eggs, which is equivalent to 6% of the eggs produced by cages provided an evening ovipositional resource. Eggs from cages provided only a mid-afternoon ovipositional resource were spread over the entire 4 hr period. Sixty-seven percent were deposited in the first 2 h, and approximately 24% were deposited between 1400 and 1500 h. The remaining 9% were deposited in the last hr. Two hundred and three eggs were oviposited between 1200 and 1600 h in cages previously exposed to an ovipositional resource the evening prior.

**Table 1:** Mid afternoon egg output by *Anopheles gambiae *as influenced by previous access to an ovipositional resource.

**Treatment**		**Total eggs per cage per period**	
With ovipositional resource the previous evening		1700 – 1200 h	1200 – 1600 h
		
Cage No.	Individuals/cage		
1	14	252	0
2	18	363	0
3	17	77	0
4	16	512	1
5	20	790	10
6	20	818	38
7	20	567	154
8	20	872	0
9	19	1888	0
10	20	1855	0
11	20	1251	0
12	20	1793	0
Total	224	11038	203
		
Without ovipositional resource the previous evening			
		
Cage No.	Individuals/cage		
1	14	-	3
2	18	-	9
3	15	-	0
4	18	-	161
5	20	-	0
6	20	-	128
7	20	-	0
8	20	-	51
9	18	-	6
10	20	-	184
11	18	-	99
12	20	-	0
Total	221	-	641

## Discussion

*A. gambiae *deposits eggs in two ovipositional pulses per 24 h. Both laboratory-strain individuals and house-collected groups of *A. gambiae *showed a large ovipositional pulse that commenced at scotophase, and peaked 1–2 h later. These results agree with those of Haddow and Ssenkubuge [12] and Sumba et al. [14]. In our work with both the laboratory strain and house-collected strain, we also observed a second smaller ovipositional pulse a few hours after the first pulse. The second pulse by laboratory strain groups occurred between 0 and 1100 h, while this second pulse occurred between 500 and 1300 h for the house-collected strain. For individuals, the onset of the second pulse occurred earlier than its occurrence in the group tests (Figure 5). Most eggs were deposited between 2300 and 0 h.

Between the first and second ovipositional pulses in all groups, egg deposition sharply declined. While both laboratory and house-collected strains decreased ovipositional activity at 0 h, laboratory strain egg deposition resumed at 100 h, while oviposition by house-collected females remained sparse until 600 h. We speculate that the significant midnight decline in egg deposition may be the result of an endogenous rhythm. The length of the quiescent period between pulses may be a direct result of exposure to certain environmental conditions, such as early morning low temperatures probably experienced by house-collected, but not laboratory strain females during these tests.

Jones and Gubbins [20] reported that peak flight by *A. gambiae *occurs immediately after lights off and that a second smaller peak in activity occurs between 6 and 10 h later. This suggests that flight activity is regulated by a circadian rhythm that could secondarily influence ovipositional patterns [20,21]. Increasing flight activity during the onset of scotophase would increase the probability that a female encounters a suitable ovipositional resource. These peak flight times described by Jones et al. [21] and Jones and Gubbins [20] may contribute to the dusk and early morning peaks in oviposition that we have recorded.

Our research established that individual females rarely split their eggs over two distinct time periods but rather lay eggs steadily after oviposition begins. We conclude that the two pulses in oviposition by groups are not the result of individual females spreading their eggs over two distinct time periods. Instead, some individual females delay the onset of oviposition to create the second peak. There was much greater variability in the preoviposition interval (i.e., the time interval prior to when a female initiated oviposition) than there was in the amount of time devoted to oviposition. In the case of the single female who split her eggs between two ovipositional periods, an interruption caused by the exchange of the paper towelling could explain this single aberration.

While the rates of egg deposition by gravid and hypergravid females were not found to be different, a statistically significant correlation existed between the length of the preoviposition interval and the total number of eggs deposited per female. Individuals with longer preoviposition intervals tended to deposit slightly more eggs. However, this correlation likely has little biological significance due to the considerable scatter in the data. This is demonstrated by the width of the 95% confidence intervals surrounding the mean total numbers of eggs per preoviposition interval, which ranged from a mean of 64.4 ([90.3, 154.72] eggs with a preoviposition interval of 14 h) to a mean of 19.9 ([80.1, 100] eggs with a preoviposition interval of 4 h).

*A. gambiae *does have the capacity for afternoon oviposition in full light. Females denied an ovipositional resource for 18 h oviposited between 1200 and 1600 h, when the ovipositional resource was introduced. In some cases, eggs were found on the ovipositional resources between 1200 and 1600 h even when the mosquitoes had a resource beginning at 1700 h on the previous night.

Visual contrast of the ovipositional substrate is an important stimulus for oviposition and egg placement. Huang et al. [18] reported that a black ovipositional dish on a white or grey floor received many more eggs than any other white-black or grey-black combination of ovipositional substrate and background. Clay soil in Kisumu, Kenya, appears black when wet and grey when dry, and discrimination between grey and black coloration improves at light levels of 2.1 × 10^-3 ^w m^-2^, which is equivalent to late dusk or early dawn [18]. When ovipositional resources are sparse, it may benefit *A. gambiae *to forage for ovipositional sites before full darkness and at or after dawn, when useful visual contrast would be more detectable.

Our overall results establish that oviposition by *A. gambiae *is not restricted only to one specific time of day, and oviposition is not fully inhibited by high light levels. Gravid females can initiate oviposition as soon as an ovipositional resource becomes available. Thus, ovitraps, a tool to monitor *A. gambiae *population growth and help predict malaria epidemics, should remain available throughout the full 24 hr diel to be maximally effective. Further study of abiotic factors like daily temperature and relative humidity fluctuations and their contribution to patterns in flight activity in the field may be of interest. Excess temperature and low RH may limit mid-afternoon oviposition in the field. During the daytime in the tropics, air and soil temperatures typically exceed the optimum temperature for oviposition (< 25°C, Huang et al. unpublished data).

## Conclusion

*A. gambiae *populations are ovipositionally flexible. Rather than confining oviposition to a specific brief period during 24 h, as is true for many insects, *A. gambiae *can oviposit at any time after their eggs have fully developed and they have access to an ovipositional resource. But, they most commonly begin oviposition and deposit the majority of eggs shortly after lights off. Once oviposition commences, individual females deposit their eggs over a continuous 2 to 3 hr period without interruption.

## Competing interests

The author(s) declare that they have no competing interests.

## Authors' contributions

MLF conducted experimental work on individuals, and primarily developed the manuscript. JH, MLF and JRM collectively planned the study and conducted experimental work with groups. JRM initiated the study and designed the clock apparatus. JRM and JH developed the manuscript with MLF. MLF, JRM, JH and EDW analyzed and displayed the data. EDW secured the NIH grant under which this research was funded, arranged Kenya travel and secured the lab facilities for the study. MNB provided logistical support, research site security and interpretational analysis. JV provided research site security and secured personnel for collection of feral mosquitoes. All authors read and approved the final manuscript.
